# Lower Extremity Strength and Range of Motion in High School Cross-Country Runners

**DOI:** 10.1155/2018/6797642

**Published:** 2018-08-08

**Authors:** Jun G. San Juan, David N. Suprak, Sean M. Roach, Marc Lyda

**Affiliations:** ^1^Department of Health and Human Development, Western Washington University, 516 High St., MS 9067, Bellingham, WA 98225, USA; ^2^Western Institute of Neuromechanics, 244 E. Broadway, Eugene, OR 97401, USA

## Abstract

Cross-country running is becoming an increasingly popular sport, with a significant participation noted at the high school level. The aim of this study was to compare gender and bilateral hip extension range of motion and hip and knee extension strength of high school cross-country runners. 31 participants volunteered from a local high school cross-country team (16 males and 15 females). The modified Thomas test was utilized to measure hip extension range of motion bilaterally using a digital inclinometer. In order to measure hip and knee isometric strengths, an isokinetic dynamometer was employed. A mixed model approach revealed a statistically significant difference in peak hip extension strength between genders but not the side. Male athletes demonstrated a 29.2 Nm/kg (*P* < 0.05) greater force production than females during isometric hip extension strength testing. There were no significant differences in peak knee extension isometric strength, hip extension range of motion, and the ratio of peak hip and knee strength between genders and the dominant and nondominant leg. Female cross-country runners should focus on increasing hip extension strength to help maintain hip stability during running. This may be beneficial in decreasing the chances of experiencing patellofemoral pain in long-distance runners.

## 1. Introduction

Running is becoming an increasingly popular activity in the United States, with a significant participation noted at the high school level. According to the National Federation of State High School Associations, there were over 471,000 athletes who participated in high school cross-country in 2014, marking a 30% increase in participation over the past 10 years [1]. The increase in participation as well as increased frequency of training can lead to significantly increased exposure to running-related injuries (RRI), most notably in the lower extremities [2]. Several recent studies noted lower extremity injury rates in high school cross-country participants ranging from 19% to 79% and up to 92% when all regions of the body were included [3]. The most common RRIs are reported to be patellofemoral pain syndrome, lower back pain, iliotibial band syndrome, plantar fasciitis, and lower leg injuries, followed by issues with the lower leg, Achilles/calf, and heel anatomy [4].

Patellofemoral pain (PFP) is a common orthopedic problem in our society, accounting for up to 25% of all knee problems addressed in sports medicine centers [4, 5]. The presence of PFP can result in not only loss of participation in activity but also significant frustration when seeking appropriate diagnosis and management of the condition. Current management strategies for the condition have been met with limited success. The literature on PFP has recently focused attention on the importance of hip strength, in particular, the hip abductors and external rotators [6, 7]. Ireland et al. [6] have demonstrated that active females with PFP have significant weakness in the hip muscles, which may lead to an alteration in lower extremity mechanics. Additionally, Powers [8] has noted that most of the lower extremity injuries experienced with athletic participation could be the result of the same phenomena, which is excessive hip internal rotation combined with adduction during the stance phase of activity. This pronation of the hip is required for proper alignment of the leg segments for stability and appropriate shock attenuation [7]. Further, it has been shown that PFP patients exhibited a decreased passive hip extension range of motion [9].

The importance of eccentric control of hip adduction during activity has been discussed as a factor in influencing the health and function of the patellofemoral joint in particular [10]. A decrease in control of eccentric hip adduction during activity results in an excessive internal rotation of the femur, with the tibia in relative external rotation, resulting in abnormal valgus stress at the anterior medial knee joint, in addition to potentially excessive loading of the hip into internal rotation and the lower leg into excessive pronation [11, 12]. It has, therefore, been suggested that interventions for PFP should include a focus on influencing stability of the hip and the pelvis to control for this excessive valgus stress at the knee [13–15].

Previous literature had noted several biomechanical differences between male and female runners. The variables most often examined include hip and knee kinematics, muscle activation, and muscle strength. In particular, it has been demonstrated that female runners exhibit greater peak hip adduction and internal rotation and knee abduction angles as compared to males [16]. This includes both healthy runners and those with either PFP or iliotibial band syndrome [17, 18]. In addition, clear differences are noted in both hip strength and muscle activation between the genders [19–21].

It is clear that hip kinematics and strength are related to the health and function of the patellofemoral joint, and that runners with certain biomechanical characteristics are susceptible to faulty mechanics at this, and surrounding, joints. It is also known that male and female runners exhibit different hip and knee kinematic patterns during running gait. However, there currently exists a paucity of normative data pertaining to hip and knee kinematics and strengths in developing male and female runners. Therefore, the purpose of the study was to compare bilateral hip extension range of motion and hip and knee extension strengths of high school runners before the start of the season. Additionally, we wanted to compare these variables between genders. We hypothesized that there would be no statistically significant difference between genders in hip extension range of motion and hip and knee strength bilaterally.

## 2. Materials and Methods

The current study was conducted at the Biokinesiology Laboratory at Western Institute of Neuromechanics in Eugene, Oregon. A total of 31 participants (age: 15.6 ± 1.2 y/o, height: 169.1 ± 12.2 cm, mass: 58.3 ± 8.8 kg) were recruited from a local high school cross-country team (16 males and 15 females). A priori estimate of the sample size revealed that a total of 26 participants were needed to achieve a power of 0.8 with an alpha level of 0.05. The study was approved by the Western Washington University human participants review committee (#12-013). Before data collection, informed consent was obtained from all participants and parents/guardians filled out the assent forms for minors.

Participants were asked to come into the laboratory one time in which all testing was completed. During the testing, participants did a five-minute warm-up on a Schwinn Airdyne (Nautilus, Vancouver, WA, USA) upright exercise bike. After the warm-up, participants had hip extension (EXT) measurement on the dominant and nondominant hips. This ROM was measured using a digital inclinometer (Digital Protractor Pro 3600, Mitotoyo America, Aurora, IL, USA). The use of this instrument has been previously validated and was reported to have a reliability of 0.9 [22]. To measure hip extension ROM, the participants lay supine on a treatment table and a modified Thomas test was performed [23]. This maneuver was implemented with the participant holding their contralateral leg with the hip and knee flexed against their chest while the ipsilateral leg being measured hung over the end of the treatment table. The investigator made sure that the hip was not in an abducted position. Further, the investigator provided both verbal and tactile cues to maintain their lower back flat against the table to avoid lumbar extension and pelvic tilting during the evaluation. The inclinometer was positioned on the anterior aspect of the thigh at the midpoint between the greater trochanter and the lateral epicondyle of the femur. All of the measurements for ROM, on the dominant and nondominant hips, were taken three times. An average was calculated for data analysis, and all the measurements were randomized.

Participants then underwent isometric strength testing using a Biodex System 3 Isokinetic dynamometer (Biodex Medical Systems, Shirley, NY, USA). Hip extension and knee extension strengths were measured on both legs for each participant. Once again, the order was randomized to determine if testing would first be done on hip extension or knee extension and then if the dominant or nondominant leg would be tested first. To measure hip extension strength, participants were asked to stand in front of the Biodex chair while flexing their trunk to 45° with respect to the ground and with the tested hip flexed to 30°. Participants were instructed to grasp the chair handles for support. The thigh of the tested leg was secured distally above the medial and lateral epicondyle using the thigh attachment that came with the dynamometer. The contralateral foot was kept flat on the ground. The manufacturer's recommended protocol was utilized for isometric knee extension strength testing. The knee was positioned at 60° of flexion during the isometric testing, and the knee attachment was secured in the distal shank immediately superior to the malleoli. In each test position, participants performed a maximum voluntary isometric contraction (MVIC) for 5 seconds three times, and the average of the three trials was utilized for analysis. Participants were given a minute rest in between testing positions to avoid fatigue. All the strength data were normalized to participant's body weight to account for differences in subjects' stature.

A two-way mixed analysis of variance (ANOVA) was used to examine the effects of the gender and side (dominant versus nondominant) on each dependent variable of interest, which included normalized peak hip extension strength, normalized peak knee extension strength, the normalized peak hip/knee extension strength ratio, and modified Thomas test range of motion. Simple effects analyses were conducted in the event of a significant gender by side interaction. The significance level for all statistical analyses was set at *P* < 0.05.

## 3. Results and Discussion

**Figure 1:**
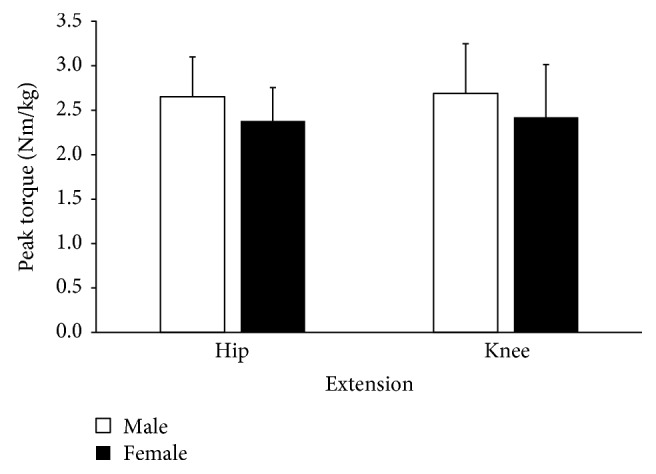
Hip and knee extension peak isometric torque between genders.

The two-way mixed ANOVA revealed no significant side by gender interaction effect on peak hip extension strength (*F*[1, 29] = 0.059, *P* = 0.81, *η*^2^ = 0.002). In addition, there was no significant main effect of the side on peak hip extension strength (*F*[1, 29] = 0.518, *P* = 0.48, *η*^2^ = 0.018). However, peak hip extension strength (Figure 1) was significantly higher in males than in females across both sides (*F*[1, 29] = 5.96, *P* = 0.02, *η*^2^ = 0.171).

The two-way mixed ANOVA revealed no significant interaction effect between gender and side (*F*[1, 29] = 2.63, *P* = 0.12, *η*^2^ = 0.083) on peak knee extension strength, as well as no main effects of neither gender (*F*[1, 29] = 1.19, *P* = 0.28, *η*^2^ = 0.04) nor side (*F*[1, 29] = 1.09, *P* = 0.31, *η*^2^ = 0.036).

As with the effects of gender and side on peak knee extension strength, the ratio between the peak hip and knee extension strengths was not significantly affected by the interaction between gender and side (*F*[1, 29] = 0.677, *P* = 0.42, *η*^2^ = 0.023), nor by neither of the main effects of gender (*F*[1, 29] = 0.224, *P* = 0.64, *η*^2^ = 0.008) nor side (*F*[1, 29] = 0.002, *P* = 0.96, *η*^2^ < 0.001).

According to the two-way mixed ANOVA, Thomas test range of motion (Table 1) was not affected significantly by the interaction of gender and side (*F*[1, 29] = 2.72, *P* = 0.11, *η*^2^ = 0.086), nor by neither of the main effects of gender (*F*[1, 29] = 0.685, *P* = 0.42, *η*^2^ = 0.023) nor side (*F*[1, 29] = 2.21, *P* = 0.15, *η*^2^ = 0.071).

The primary purpose of this study was to compare the differences in bilateral hip and knee extension strengths and hip extension mobility between high school cross-country runners. Additionally, the ratio of the average peak isometric strength of the knee extensors and hip extensors was compared between genders. The results of the current study support the hypothesis that there will be no statistically significant differences between gender and side in both the hip and the knee range of motion and strength except for the peak hip extension strength. The current data indicated that males demonstrated significantly larger peak hip extension strength compared to females. This area of exploration was conducted in order to assist in further insights into common RRI such as PFP and iliotibial band syndrome (ITBS).

It has been increasingly documented that gluteal muscle strength is an important variable in control of the femur during functional tasks such as running and stair climbing and descent [18, 20, 21]. The gluteus maximus main action is to extend the hip joint, and it also plays a vital role in running by eccentrically controlling the hip during internal rotation in upright tasks [24]. Notably, evidence shows that individuals with PFP have increased hip internal rotation in running as compared to controls [20]. Souza and Powers [20] hypothesize that it is the increased internal rotation of the femur relative to the tibia that creates a lateral positioning of the patella, and thus increased loading of the retro patellar region.

The present study was conducted to examine the difference between genders in hip and knee extension strengths. Willson et al. [21] found that healthy female runners exhibited a 40% greater peak gluteus maximus activation level compared to male runners. Additionally, Souza and Powers [20] noted an increase in gluteus maximus activation in female runners with PFP as opposed to female controls. The accumulating evidence lends credence to the hypothesis that the increased gluteus muscle activation in females with PFP, and even those without, may lead to earlier fatigue of the muscle and thus impaired control of frontal and transverse plane hip motion [20, 21]. The results of the current study revealed a marked difference in peak hip extension strength between male and female runners. A combination of decreased overall strength and possible decreased endurance of the gluteus maximus in runners, particularly females, provides valuable information for better-designed conditioning and rehabilitation programs for runners. This decreased hip strength observed in high school cross-country runners may lead to early fatigue and impaired lower extremity kinematics. Runners may benefit from both aggressive isolated hip extensor muscles strengthening (i.e., gluteus maximus) and endurance-type training to help normalize lower extremity mechanics during running activities.

In addition, females exhibited significantly lower peak hip strength values than males did, but no differences were noted in knee extensor values between gender and sides. Males exhibited 29.2 Nm/kg greater force production during hip extension. Of most interest is that no significant differences were noted in knee-hip extensor strength ratios between groups. With regard to RRI, there is strong evidence to support that females tend to use a more quadriceps- or knee extensor- dominant pattern with landing activities [25]. Stearns and colleagues [26] have documented that women tend to demonstrate a higher knee-hip extensor ratio than men during the deceleration phase of landing. One reason for the difference in findings could be the result of comparing a double leg drop jump task versus running. Although running certainly involves a deceleration phase during the landing on one limb, increased control strategies may differ with double leg activities.

The current results did not show a difference in hip extension mobility when compared bilaterally and between genders. This variable is important to help better understand the influence of altering hip mobility in the sagittal plane, which could provide clues to differences in gender-specific injury rates. Given the importance of the gluteus maximus to function within a full range of motion, it was important to determine if a lack of mobility existed with this population, since recent evidence demonstrated a difference in subjects with PFP and controls [9]. Roach and colleagues [22] compared hip mobility in healthy and PFP subjects. The authors found out that PFP patients experienced decreased hip extension ROM compared to controls. The combined gender average hip extension of runners in the current study was 6.6° on the nondominant and 7.7° on the dominant leg, which was consistent with that of the Roach et al. [9] study on healthy active individuals of 6.8° bilaterally.

There were a few limitations in this current study. First was the fact that the participants were uninjured, and therefore, the findings may not be representative of injured runners. It may be valuable to investigate the differences between healthy runners and injured runners to determine if these observations are consistent. The second limitation was that the study examined isometric strength and did not look at strength through a full dynamic range of motion. Future studies should examine the same variables within an injured population of high school-aged runners for comparison.

## 4. Conclusion

This study observed a significant difference in hip extension strength between genders in healthy high school cross-country runners. Males exhibited a higher hip extensor strength when tested isometrically than the female participants. However, it is worth noting that there are no significant differences observed in the knee-hip extension strength ratios and hip extension range of motion between genders. The results also presented that high school female cross-country runners do not exhibit a greater knee extensor strength when compared to their hip extensors. The results observed in the current study are all factors that can contribute to decreasing or preventing the chance of suffering a running-related injury in high school cross-country runners.

## Figures and Tables

**Table 1:** Hip extension range of motion measured during the modified Thomas test (mean ± SEM) between gender and the side (i.e., the dominant and nondominant leg).

Hip extension range of motion (°)		
Gender	Dominant	Nondominant
Male	5.9 ± 1.5	6.0 ± 1.5
Female	9.6 ± 2.5	7.1 ± 1.8

## Data Availability

The data used to support the findings of this study are available from the corresponding author upon request.
